# Integrated Transcriptome and Metabolome Analysis Reveals Molecular Mechanisms Underlying Resistance to *Phytophthora* Root Rot

**DOI:** 10.3390/plants13121705

**Published:** 2024-06-19

**Authors:** Ruidong Sun, Anan Han, Haitang Wang, Congcong Wang, Yang Lu, Danqing Ni, Na Guo, Han Xing, Jinming Zhao

**Affiliations:** Key Laboratory of Biology and Genetics and Breeding for Soybean, Ministry of Agriculture, Zhongshan Biological Breeding Laboratory, National Innovation Platform for Soybean Bio-Breeding Industry and Education Integration, State Key Laboratory of Crop Genetics & Germplasm Enhancement and Utilization, College of Agriculture, Nanjing Agricultural University, Nanjing 210095, China; 2018201070@njau.edu.cn (R.S.); 2021101136@njau.edu.cn (A.H.); 2012093@njau.edu.cn (H.W.); 2018201069@njau.edu.cn (C.W.); 2021201071@stu.njau.edu.cn (Y.L.); 2021101135@njau.edu.cn (D.N.)

**Keywords:** *Glycine max*, defense response, *Phytophthora sojae*, gene expression profile, plant–pathogen interaction

## Abstract

Soybean production is significantly impacted by *Phytophthora* root rot (PRR), which is caused by *Phytophthora sojae*. The nucleotide-binding leucine-rich repeat (NLR) gene family plays a crucial role in plant disease resistance. However, current understanding of the function of soybean *NLR* genes in resistance to PRR is limited. To address this knowledge gap, transgenic soybean plants overexpressing the *NLR* gene (*Glyma.18g283200*) were generated to elucidate the molecular mechanism of resistance. Here, transcript changes and metabolic differences were investigated at three time points (12, 24, and 36 h) after *P. sojae* infection in hypocotyls of two soybean lines, Dongnong 50 (susceptible line, WT) and *Glyma.18g283200* overexpression line (resistant line, OE). Based on the changes in differentially expressed genes (DEGs) in response to *P. sojae* infection in different lines and at different time points, it was speculated that HOPZ-ACTIVATED RESISTANCE 1 (ZAR1), valine, leucine, and isoleucine degradation, and phytohormone signaling may be involved in the defense response of soybean to *P. sojae* at the transcriptome level by GO term and KEGG pathway enrichment analysis. Differentially accumulated metabolites (DAMs) analysis revealed that a total of 223 and 210 differential metabolites were identified in the positive ion (POS) and negative ion (NEG) modes, respectively. An integrated pathway-level analysis of transcriptomics (obtained by RNA-seq) and metabolomics data revealed that isoflavone biosynthesis was associated with disease resistance. This work provides valuable insights that can be used in breeding programs aiming to enhance soybean resistance against PRR.

## 1. Introduction

*Phytophthora* root rot (PRR), caused by *Phytophthora sojae*, is a major global disease of soybean. The causal fungus is a polycyclic pathogen that leads to huge yield losses [1,2]. Host resistance is both economical and environmentally friendly to prevent yield loss and quality degradation [3]. Therefore, the development of PRR-resistant soybean varieties is an important task to control this disease.

Two strategies have been identified in plants to defend against pathogen infection, known as pattern-triggered immunity (PTI) and effector-triggered immunity (ETI). PTI provides a first line of defense and is thought to be effective in preventing most potential pathogens. Cell surface-localized pattern recognition receptors (PRRs) recognize pathogen/microbe-associated molecular patterns (PAMPs/MAMPs) from pathogens to activate defenses [4]. ETI is activated by the recognition of microbial effectors by cytoplasmic nucleotide-binding leucine-rich repeat (NLR) receptors [5]. To date, with the development of DNA sequencing technology, nearly 40 genes conferring resistance to *P. sojae* (*Rps*) have been identified, and most *Rps* genes are predicted to encode NLR proteins [2,6]. A giant *NLR* gene is identified as the candidate gene of *Rps11* by fine mapping and expression analysis [7]. *RpsGZ* has been mapped to a 367.371 kb genomic region on chromosome 3 that was predicted to contain seven *NLR* genes [3]. The activation of ETI usually triggers multiple downstream responses, such as the generation of reactive oxygen species (ROS), cell wall reinforcement, and the biosynthesis of antimicrobial compounds [8]. Nevertheless, the mechanisms of molecular responses mediated by *Rps* genes remain largely unknown.

RNA sequencing (RNA-seq) is an essential tool for elucidating plant–pathogen interactions, especially in gene expression analysis, candidate resistance gene discovery, and the identification of disease-resistance pathways [9]. A few transcriptomic and metabolomic studies also uncovered molecular pathways in response to *P. sojae* infection. Comparative transcriptome analysis of resistant near-isogenic lines (NILs) and susceptible control revealed ethylene, jasmonic acid, MAPK regulatory networks, and ROS underlying the defense responses [10]. By studying the differential gene expression patterns in soybean during infection by *P. sojae*, it was found that most of the up-regulated cDNAs encode pathogenesis-related proteins and enzymes of phytoalexin biosynthesis [11]. In addition, metabolomic analysis hypothesized that sugars, organic acids, amino acid derivatives, and other secondary metabolites such as daidzein, octanal, hypoxanthine, and mannitol may be involved in the defense response of soybean to *P. sojae* at the metabolic level [12].

Transgenic technology has resulted in an elite soybean variety with resistance to *P. sojae* by transferring specific pathogen resistance genes from resistant varieties into cultivated soybeans [7]. The molecular processes underlying the host–pathogen interaction in plants would be better understood using transgenic lines. The southern arm of chromosome 18 is an *Rps* gene cluster containing *Rps4*, *5*, *6*, *12*, *13*, and *JS*, and several *NLR* genes in this interval are thought to be associated with PRR resistance, such as *Glyma.18g51950*, *Glyma.18g51960*, and *Glyma.18g283200* [13,14]. Previously, stable *P. sojae*-resistant transgenic soybean plants were generated by overexpressing *Glyma.18g283200* in the *P. sojae*-susceptible Dongnong 50 [15]. To elucidate the molecular mechanism of the *NLR* gene in enhancing soybean resistance to PRR and to discover novel disease resistance metabolites and their corresponding regulatory genes, RNA-seq and metabolite accumulation analysis were performed in a transgenic resistant soybean line (OE) containing the *NLR* gene and wild-type Dongnong 50 (WT) in response to *P. sojae* infection. GO term and KEGG pathway enrichment analysis were performed to reveal the effect of the NLR on soybean resistance to *P. sojae* infection. The integrated pathway-level analysis of transcriptomics and metabolomics data were used to explore the mechanism of improved PRR resistance in soybean overexpressing the *NLR* gene.

## 2. Results

### 2.1. Overview of RNA Sequencing Data

To understand the mechanisms of the effects of *Glyma.18g283200* during *P. sojae* infection, deep transcriptome sequencing analysis of WT and OE was performed at 12 h, 24 h, and 36 h after inoculation with *P. sojae* (Figure S1 and Figure S2). The sequencing quality for all samples was quite high after filtering the raw sequencing data. The percentage of bases with Q20 (high sequencing quality) was nearly 100% (Figure S3A). The gene coverage of 80% to 100% accounted for about 70% of the total genes (Figure S3B). Summaries of the raw sequence quality before and after filtering and the read counts mapped to the reference genome (*Glycine max* Wm82.a4.v1) are detailed in Supplementary Table S1. Among the high-quality clean reads obtained from the 18 samples, the percentage of total mapped reads was 95.14–97.34% (Table S1). These results indicate that the quality of the transcriptome sequencing was sufficient for further analysis. The samples were clustered using principal component analysis (PCA) and correlation analysis to determine the relationship between the three biological replicates. PCA and correlation heatmaps show excellent reproducibility between biological replicates (Figure S4A,B). The Pearson correlation coefficient for the three biological replicates of each group also showed very high reproducibility (R^2^ ≥ 0.91).

### 2.2. Differentially Expressed Genes in Response to Phytophthora sojae Infection

**Figure 1:**
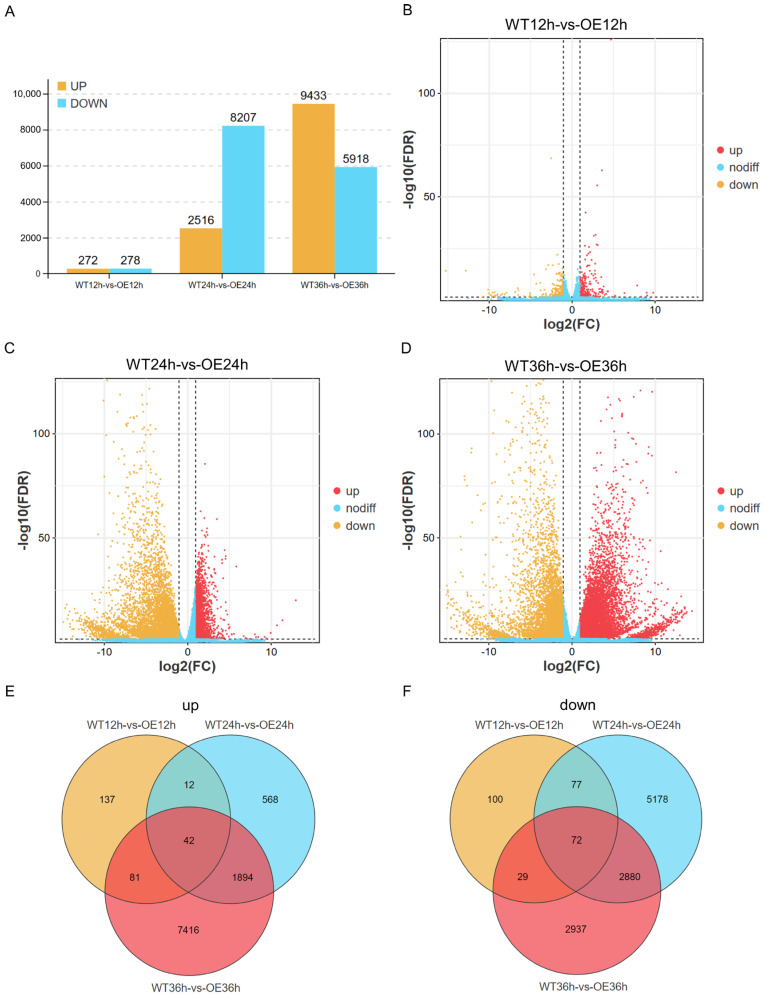
Differential analysis of gene expression between WT and OE groups. (**A**) Comparing the number of up- and down-regulated genes. (**B**–**D**) Volcano plots of differentially expressed genes at 12, 24, and 36 hpi between the WT and the OE plants as determined by RNA-seq. Red and yellow dots represent up- and down-regulated genes separately. Blue dots represent no differential genes. (**E**) Up-regulated DEGs at 12, 24, and 36 hpi compared by Venn diagram. (**F**) Down-regulated DEGs at 12, 24, and 36 hpi compared by Venn diagram.

To clarify the mechanism underlying soybean resistance to *P. sojae*, differentially expressed genes (DEGs) of WT and OE were analyzed at 12 hpi, 24 hpi, and 36 hpi. To identify the genes with differential expression between the WT and the OE plants, DEGs were determined using the R/edgeR package [16] with the following criteria: FDR < 0.05 and |log2FC| > 1. The total number of genes that were significantly regulated was different in the different comparisons (Tables S2–S4). There were 550 DEGs in WT 12 h vs. OE 12 h, including 272 up-regulated genes and 278 down-regulated genes; 10,723 in WT 24 h vs. OE 24 h, including 2516 up-regulated genes and 8207 down-regulated genes; and 15,351 in WT 36 h vs. OE 36 h, including 9433 up-regulated genes and 5918 down-regulated genes (Figure 1). These results showed that there were more genes involved in the response to *P. sojae* infection at 24 hpi and 36 hpi, among which there were many essential genes that may be more relevant to the resistance to *P. sojae*. A Venn diagram analysis showed that 42 genes were commonly up-regulated in OE compared to WT after pathogen infection, including a SODB gene (*Glyma.10G193500*) involved in peroxisome (ko04146), two SAUR-like auxin-responsive genes (*Glyma.09G219300*, *Glyma.09G221800*) involved in plant hormone signal transduction (ko04075), and an unannotated novel gene (MSTRG.27554) involved in purine metabolism (ko00230) (Figure 1E, Table S5). Additionally, there are other genes encoding protein kinases (*Glyma.10g156200*), guanine nucleotide exchange factor (*Glyma.11G225200*), expansin-B3-like precursor (*Glyma.12G203600*), receptor-like protein kinase (*Glyma.16G185100*), GDSL esterase/lipase (*Glyma.17G259600*), and a DnaJ family gene (*Glyma.01G040600*) (Figure 1E, Table S5). These genes may be involved in resistance to biotic stress. In comparison, we observed that 72 genes were commonly down-regulated in OE compared to WT following pathogen infection (Figure 1F, Table S6). Among these genes, 13 are associated with metabolic pathways (ko01100), 3 (Glyma.11G124500, Glyma.16G119200, Glyma.17G076100) are involved in amino sugar and nucleotide sugar metabolism (ko00520), and 2 genes (*Glyma.08G317100*, *Glyma.13G065400*) are related to oxidative phosphorylation (ko00190).

### 2.3. Validation of the Transcriptomic Data Based on the RT-qPCR Analysis

To assess the reliability of the transcriptome information, six DEGs were randomly selected to validate the RNA-seq data at each time point. The RT-qPCR results confirmed that the expression profiles of all selected DEGs were highly consistent with those obtained from the transcriptome data (Figure S5).

### 2.4. Functional Classification of DEGs of WT and OE Plants

**Figure 2:**
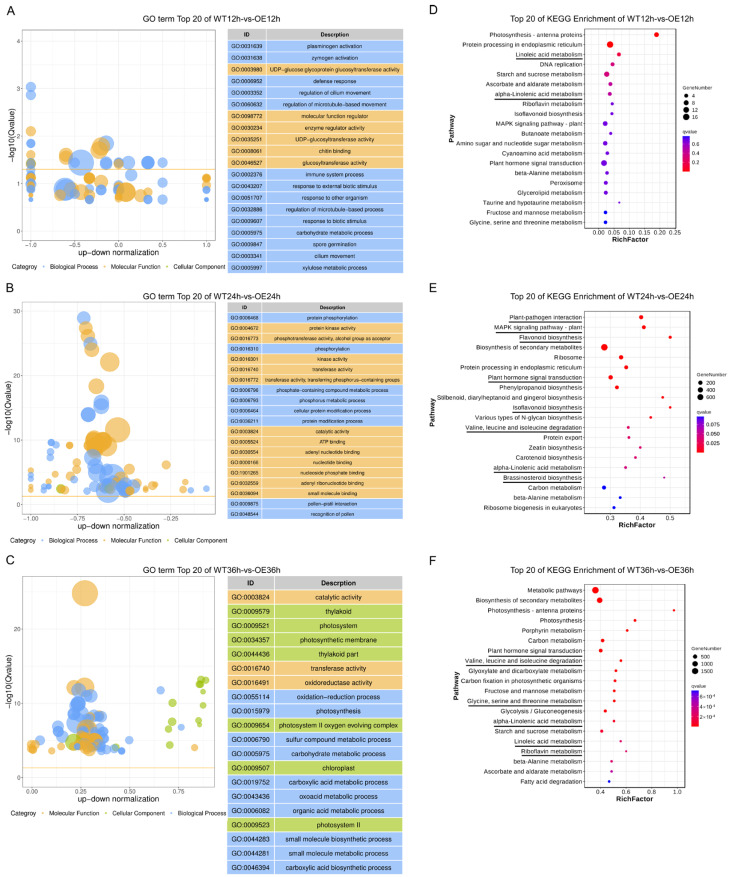
GO and KEGG enrichment analysis of DEGs between WT and OE plants after infection with *P. sojae*. The bubble chart of GO enrichment analysis of DEGs between WT and OE at 12 hpi (**A**), 24 hpi (**B**), and 36 hpi (**C**). The bubble size indicates the number of genes in the GO terms. Blue bubbles represent biological processes; orange bubbles represent molecular functions; green bubbles represent cellular components. The top 20 categories are shown on the right. KEGG enrichment analysis of DEGs between WT and OE at 12 hpi (**D**), 24 hpi (**E**), and 36 hpi (**F**). The x-axis indicates the rich factor, and the y-axis indicates the pathway name. The size of the dot represents the number of DEGs. The different colors of the dots represent different q values. Significantly enriched pathways (*p* < 0.05) associated with disease resistance are underlined.

To further explore the molecular response of WT and OE, we analyzed the functional annotation of DEGs after *P. sojae* infection (WT12h vs. OE12h, WT24h vs. OE24h, WT36h vs. OE36h). The DEGs were assigned to three GO categories: biological process, cellular component, and molecular function. In the biological process, important terms at 12 hpi were associated with defense response, immune system processes, and response to biotic stimuli, among others (Figure 2A). In molecular function, GO terms at 12 hpi were associated with chitin-binding (Figure 2A). GO enrichment analysis showed that phosphorylation-related terms were enriched at 24 hpi, including protein phosphorylation, protein kinase activity, and phosphotransferase activity (Figure 2B). GO terms associated with catalytic activity, oxidoreductase activity, and oxidation–reduction processes were enriched at 36 hpi (Figure 2C). Such terms were previously thought to be essential for disease resistance mechanisms in plants.

KEGG pathway analysis revealed that DEGs at 12 hpi were significantly enriched in the first seven pathways (*p* < 0.05), such as linoleic acid metabolism, starch and sucrose metabolism, and ascorbate and aldarate metabolism (Figure 2D, Table S7). At 24 hpi, DEGs were most highly enriched in plant–pathogen interaction, MAPK signaling, and flavonoid biosynthesis (Figure 2E, Table S8). At 36 hpi, the DEGs were enriched in porphyrin metabolism, carbon metabolism, and plant hormone signal transduction (Figure 2F, Table S9). Pathways associated with disease resistance were more enriched at 24 and 36 hpi, such as plant–pathogen interaction, MAPK signaling, and riboflavin metabolism.

### 2.5. GO Term and KEGG Pathway Enrichment Analysis of Up-Regulated DEGs

**Figure 3:**
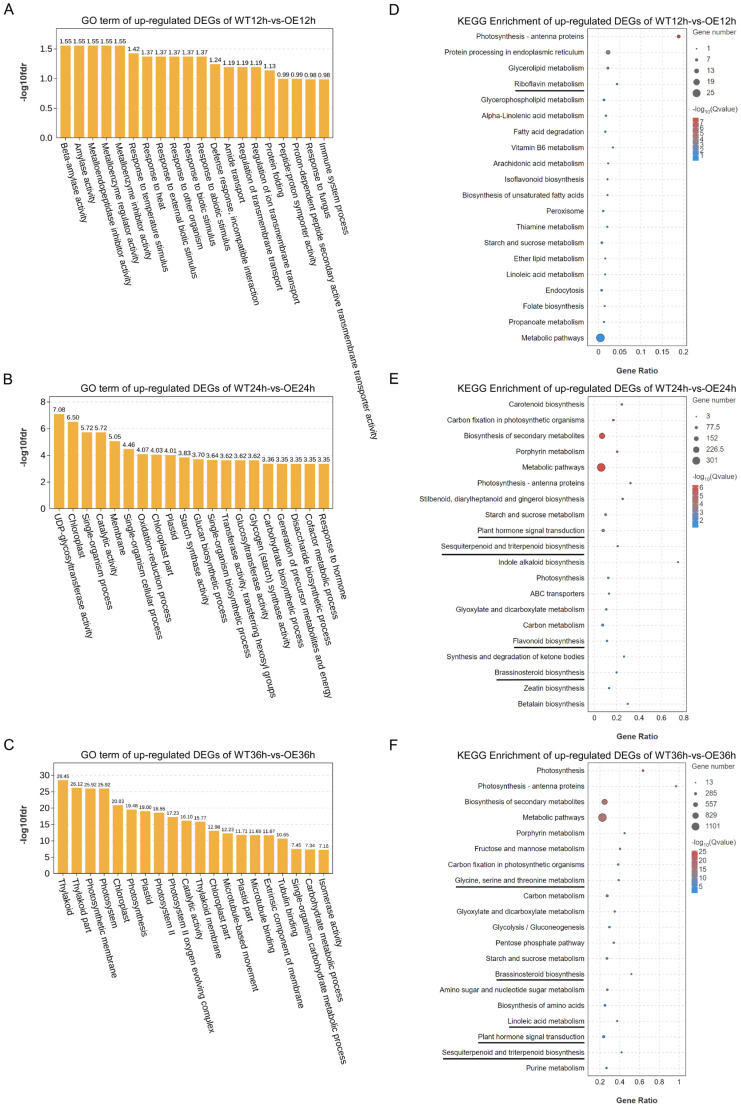
GO and KEGG enrichment analysis of up-regulated DEGs between WT and OE plants at 12, 24, and 36 hpi. (**A**) GO enrichment analysis of up-regulated DEGs between WT12h and OE12h. (**B**) KEGG enrichment analysis of up-regulated DEGs between WT12h and OE12h. (**C**) GO enrichment analysis of up-regulated DEGs between WT24h and OE24h. (**D**) KEGG enrichment analysis of up-regulated DEGs between WT24h and OE24h. (**E**) GO enrichment analysis of up-regulated DEGs between WT36h and OE36h. (**F**) KEGG enrichment analysis of up-regulated DEGs between WT36h and OE36h. Significantly enriched pathways (*p* < 0.05) associated with disease resistance are underlined.

To further understand the function of DEGs at 12, 24, and 36 hpi, all up-regulated DEGs were subjected to GO term analysis and KEGG pathway enrichment analysis (Figure 1E and Figure 3). GO term analysis demonstrated that up-regulated DEGs in WT12h vs. OE12h were primarily enriched in some biological processes (*p* < 0.05), including response to biotic stimulus (GO:0009607), defense response, incompatible interaction (GO:0009814), response to fungi (GO:0009620), and immune system processes (GO:0002376) (Figure 3A, Table S10). GO terms that were enriched in WT24h vs. OE24h were oxidation–reduction process (GO:0055114), response to hormones (GO:0009725), and UDP-glycosyltransferase activity (GO:0008194) (Figure 3B, Table S11). At 36 hpi, DEGs were most highly enriched in the thylakoid part (GO:0044436), photosynthetic membrane (GO:0034357), and chloroplast (GO:0009507) (Figure 3C, Table S12).

The results of the KEGG analysis indicated that the up-regulated DEGs in all three groups were enriched in some pathways related to resistance. Analysis of the up-regulated genes at 12 hpi revealed that the DEGs were significantly enriched in the first four pathways (*p* < 0.05), including protein processing in the endoplasmic reticulum, glycerolipid metabolism, and riboflavin metabolism (Figure 3D, Table S13). The up-regulated DEGs at 24 hpi were enriched in plant hormone signal transduction, flavonoid biosynthesis, and brassinosteroid biosynthesis (Figure 3E, Table S14). At 36 hpi, the DEGs were enriched in disease resistance-related pathways such as glycine, serine, and threonine metabolism and sesquiterpenoid and triterpenoid biosynthesis, in addition to brassinosteroid biosynthesis and plant hormone signal transduction (Figure 3F, Table S15).

### 2.6. Analysis of Gene Expression Patterns and Clustering and Functional Classification of DEGs

**Figure 4:**
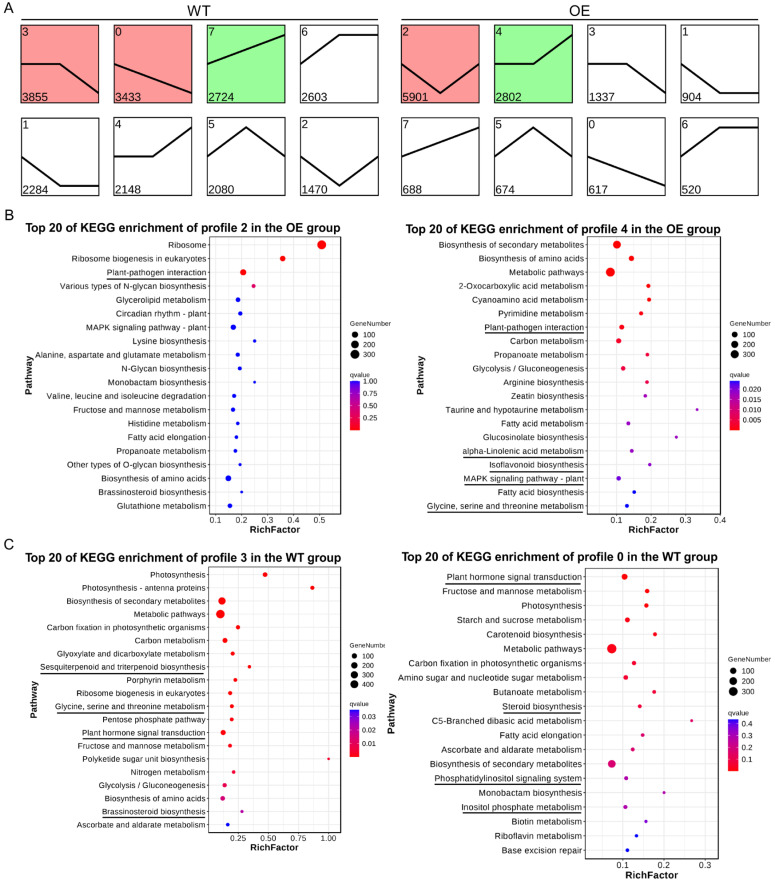
Dynamic transcriptome of WT and OE plants during *P. sojae* infection. (**A**) Gene expression patterns derived from short time-series expression miner (STEM) analysis. The number of genes in each profile is labeled in the lower left corner. The profiles are ordered based on the *p*-value significance of the number of genes assigned versus expected. The colored square frame indicates significant profiles (*p*-value < 0.05). The black line represents the overall trend in each profile. The x-axis represents stages. From left to right, they represent 12 hpi, 24 hpi, and 36 hpi. The y-axis represents the log2-fold change in gene expression, log2 (fold-change) ≥ 1. (**B**) KEGG enrichment analysis of significantly enriched profiles in the OE group. (**C**) KEGG enrichment analysis of significantly enriched profiles in the WT group. Significantly enriched pathways (*p* < 0.05) associated with disease resistance are underlined.

To understand the dynamics of the soybean transcriptome in response to *P*. *sojae* infection, a Short Time-series Expression Miner (STEM) analysis was performed on the total DEGs [17]. All sixteen temporal gene expression profiles are shown (Figure 4A). In the WT group, 48.6% (10,012) of the DEGs were clustered into three overrepresented profiles (0, 3, and 7; *p* < 0.05), of which two profiles (0 and 3) showed a trend of down-regulation after *P*. *sojae* infection (Figure 4A). In the OE group, 64.7% (8703) of the DEGs were significantly clustered into two overrepresented profiles (2 and 4; *p* < 0.05) after *P*. *sojae* infection (Figure 4A).

In the OE group, profile 2 contained the most transcripts (5901). The expression pattern of profile 2 showed a decrease at 24 hpi, followed by an increase at 36 hpi. Ultimately, there was no significant difference in the expression levels between 12 and 36 hpi. Many transcripts in profile 2 were involved in controlling the ribosome, plant–pathogen interaction, various types of N-glycan biosynthesis, and other metabolic pathways (Figure 4B, Table S16). Most of the genes in profile 4 were involved in plant–pathogen interaction, isoflavonoid biosynthesis, the MAPK signaling pathway, and glycine, serine, and threonine metabolism, and these genes were up-regulated at 36 hpi, indicating that these genes would respond to *P. sojae* infection (Figure 4B, Table S17). In addition, linoleic acid metabolism, alpha-linolenic acid metabolism, terpenoid backbone biosynthesis, and metabolic pathways were overrepresented in profile 7, and genes involved in sesquiterpenoid and triterpenoid biosynthesis, linoleic acid metabolism, steroid biosynthesis, flavonoid biosynthesis, terpenoid backbone biosynthesis, and diterpenoid biosynthesis were enriched in profile 6 (Figure S6). These transcripts were up-regulated at 24 hpi and had increased or maintained at high expression levels at the later time points sampled, suggesting that these defense pathways are continuously induced by *P. sojae* infection.

In the WT group, however, genes involved in plant hormone signal transduction, carotenoid biosynthesis, butanoate metabolism (ko00650), steroid biosynthesis (ko00100), the phosphatidylinositol signaling system, and inositol phosphate metabolism were enriched in profile 0; these genes were consistently down-regulated at 24 hpi and 36 hpi (Figure 4C, Table S18). Similarly, genes involved in sesquiterpenoid and triterpenoid biosynthesis, glycine, serine and threonine metabolism, plant hormone signal transduction, brassinosteroid biosynthesis, flavonoid biosynthesis, and terpenoid backbone biosynthesis were overrepresented in profile 3, in which gene expression was decreased at 36 hpi (Figure 4C, Table S19). This result suggests that the transcriptome response of WT to *P*. *sojae* infection would be suppressed. On the contrary, genes that were involved in the MAPK signaling pathway, valine, leucine, and isoleucine degradation, alpha-linolenic acid metabolism, carotenoid biosynthesis, plant hormone signal transduction, peroxisome, and isoflavonoid biosynthesis, which were enriched in profile 7, were up-regulated after *P. sojae* infection (Figure 4C, Table S20).

### 2.7. Differential Metabolites and Their Functional Analysis

**Figure 5:**
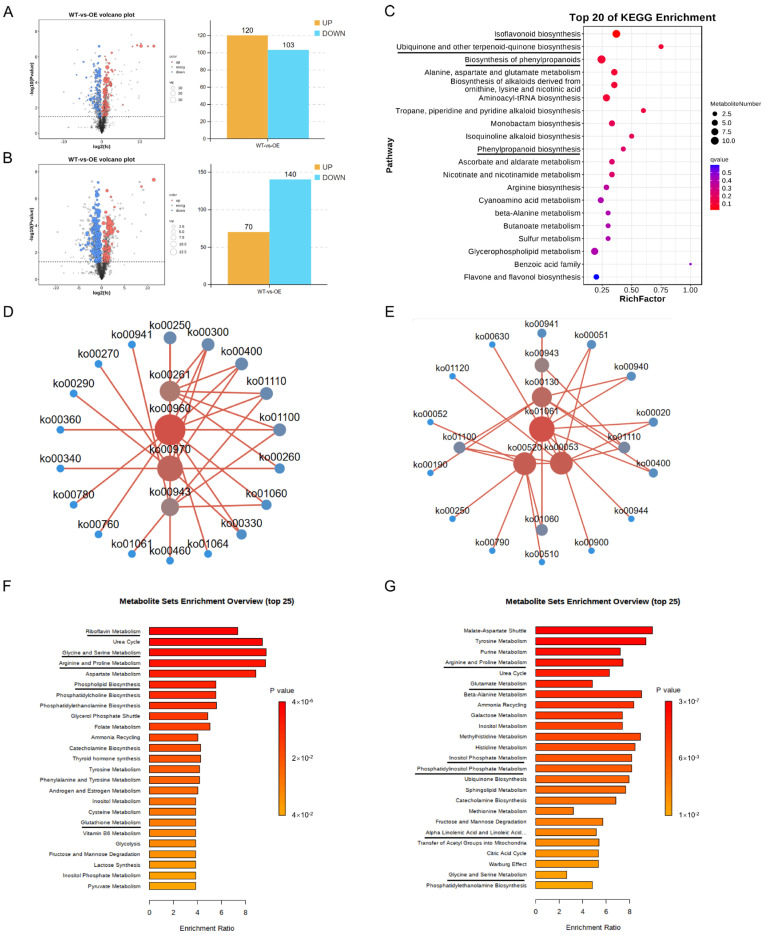
Differential metabolites and their functional analysis. (**A**) Volcanic plot and quantitative statistical plot of differential metabolites in POS. The abscissa is the value of the difference multiple of metabolite abundance in each comparison group after log2, the ordinate is the value of *P* after the *t*-test after −log10, and the dotted line perpendicular to the y-axis is the threshold of the *p*-value for differential metabolite screening. The red dots indicate the differential metabolites with VIP ≥ 1 and *p* < 0.05, which are up-regulated (FC > 1); the blue dots indicate the differential metabolites with VIP ≥ 1 and *p* < 0.05 which are down-regulated (FC < −1). The larger the dot, the higher the VIP value of the metabolite. (**B**) Volcanic plot and quantitative statistical plot of differential metabolites in NEG. (**C**) KEGG enrichment analysis of the differences in the metabolite content between the WT and OE groups. The ordinate indicates the pathway, and the abscissa indicates the enrichment factor. The size represents the number, and the redder the color, the lower the Q value. (**D**) Diagram of the KEGG pathway interaction network in POS. (**E**) Diagram of the KEGG pathway interaction network in NEG. (**F**) MSEA enrichment plot in POS. The name of the enriched metabolic set is shown on the ordinate and the degree of enrichment is shown on the abscissa. (**G**) MSEA enrichment plot in NEG. Significantly enriched pathways (*p* < 0.05) associated with disease resistance are underlined.

Metabolite accumulation analysis was further performed on the WT and OE plants in response to *P. sojae* infection at 24 h using LC-MS/MS (Vanquish, Thermo Fisher Scientific, Waltham, MA, USA) analysis. The results showed that the metabolic pattern of the WT and OE groups was altered after *P. sojae* infection and that the affected metabolites could be distinguished (Figure S7). Differential metabolites were screened with VIP ≥ 1 and *t*-test *p* < 0.05. A total of 223 differential metabolites were identified in the positive ion mode, of which 120 were up-regulated and 103 were down-regulated (Figure 5A). In the negative ion mode, 210 differential metabolites were identified, of which 70 were up-regulated and 140 were down-regulated (Figure 5B).

KEGG analysis was performed on the different metabolites and the results showed that the metabolic pathways mainly affected included isoflavonoid biosynthesis, ubiquinone and other terpenoid-quinone biosynthesis, and the biosynthesis of phenylpropanoids (Figure 5C). In addition, we mapped the network based on the interactions between different pathways in accordance with the pathway information enriched by differential metabolites. The core pathways in POS mode were tropane, piperidine, and pyridine alkaloid biosynthesis (ko00960), aminoacyl-tRNA biosynthesis (ko00970), monobactam biosynthesis (ko00261), and isoflavonoid biosynthesis (ko00943) (Figure 5D). Isoflavonoid biosynthesis is thought to play an important role in soybean resistance to *P. sojae*. The network diagram shows that the isoflavonoid biosynthesis pathway contains six differential metabolites and that the pathway interacts with five pathways (Figure 5D, Table S21). In addition to isoflavonoid biosynthesis, pathways such as the biosynthesis of phenylpropanoids (ko01061), amino sugar and nucleotide sugar metabolism (ko00520), and ascorbate and aldarate metabolism (ko00053) were also the core pathways in NEG mode (Figure 5E). The role of the phenylpropanoid pathway is important in plant defense [18]. The biosynthesis of the phenylpropanoids pathway contains six differential metabolites, and the pathway interacts with eight other pathways (Figure 5E, Table S22).

The MSEA analysis revealed that disease resistance-related pathways such as riboflavin metabolism, glycine and serine metabolism, and aspartate metabolism were enriched in the POS mode (Figure 5F). In the NEG mode, the pathways such as the malate-aspartate shuttle and tyrosine metabolism were significantly enriched (Figure 5G). In summary, OE plants enhance resistance by altering the secretion of different metabolites and interacting with the associated pathways.

### 2.8. Association Analysis of Transcriptome and Metabolome Reveals the Molecular Mechanism of OE Plants in Regulating Resistance

**Figure 6:**
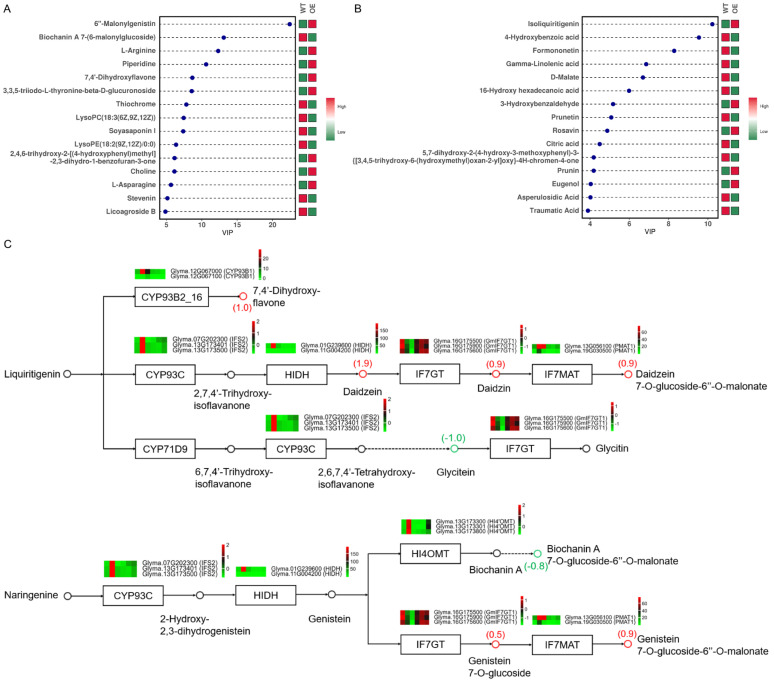
Analysis of differential metabolites and isoflavonoid biosynthetic pathways. (**A**) Top 15 differential metabolites for VIP value in the POS mode. The horizontal coordinate is the VIP value; the higher the VIP value, the greater the contribution of the metabolite to the discrimination between the WT and OE groups. (**B**) Top 15 differential metabolites for VIP value in the NEG mode. (**C**) The relative metabolite content and gene expression were integrated to construct isoflavonoid biosynthesis pathways. The red circle-labeled metabolites are up-regulated, and the green circle-labeled metabolites are down-regulated. Differential metabolites are labeled with log2FC values. The heatmaps are drawn according to the transcriptome data. The horizontal row represents a DEG with its gene ID, and the vertical columns represent WT_12 hpi, WT_24h hpi, WT_36h hpi, OE 12 hpi, OE 24 hpi, and OE 36 hpi, from left to right.

Furthermore, common pathway analysis was performed based on the enrichment of DEGs of 24 hpi and differential metabolites. In particular, isoflavonoid biosynthesis and phenylpropanoid biosynthesis were found to play important roles in resistance to *P. sojae* (Figure S8A, Table S23). Isoflavonoids constitute a category of phenylpropanoid-derived specialized metabolites found primarily in leguminous plants. Integrated transcriptome and metabolome analysis revealed that the accumulation of 6″-malonglgenistin, isoliquiritigenin, and other metabolites was higher in the OE plants, and many isoflavonoid biosynthesis-related genes were differentially expressed between the WT and OE groups (Figure 6, Table S24 and Table S25). Loading in the metabolome identified many metabolites closely related to genes which included POS00106 (saponarin), POS00084 (5-hydroxy-2-phenyl-4H-chromen-4-one), and NEG00061 (astragalin) (Figure S8B,C). Loading in the transcriptome also identified many genes related to metabolites including *Glyma.18G238100*, *Glyma.12G033900*, *Glyma.07G048800*, and *Glyma.U031505* (Figure S8B,C). In addition, differential genes and differential metabolites with correlation coefficient absolute values of >0.5 and ranks in the top 50 were plotted on the heat map (Figure S8D) and correlation network map (Figure S8E).

## 3. Discussion

Comparing the results of WT and OE plants provides a better understanding of the molecular aspects of the *NLR* gene’s strategy to respond to *P. sojae* infection. The KEGG analysis showed that a common pathway ‘valine, leucine, and isoleucine degradation’ was enriched at 24 and 36 hpi (Figure 2E,F). Previous studies have shown that supplementation with exogenous leucine results in increased resistance to *Ustilaginoidea virens* in rice. Branched-chain amino acids (BCAAs), including leucine, isoleucine, and valine, are significantly enriched in the panicles of disease-resistant rice compared with diseased rice [19]. The research showed that many genes of the valine, leucine, and isoleucine degradation pathway, such as *Glyma.11G154423*, which encodes isovaleryl-CoA dehydrogenase, were significantly up-regulated in the WT group (Figure S9A). In contrast, the expression of related genes was suppressed in the OE group (Figure S9A). The metabolomic data also showed that the levels of several amino acids were higher in the OE group, including valine (Figure S9B). Leucine suppressed the pathogenicity of *Ustilaginoidea virens* by inducing cell death through excessive H_2_O_2_ production. BCAAs also caused transcriptome reprogramming in *Ustilaginoidea virens*, thereby reducing its pathogenicity. Suppressed transcripts primarily affected processes such as effectors, pathogen–host interaction, mycotoxin biosynthesis, and hypothetical pathogenicity-associated genes [19]. It was found that treatment with 18 amino acids significantly reduced blast lesions on leaves. The treatment with amino acids increased the transcript levels of several SA-responsive genes, such as *OsWRKY19*, *OsWRKY45*, and *OsWRKY76*, as well as several JA-responsive genes, including *OsJAZ3* and *OsJAZ4* [20]. The biosynthesis of leucine and other BCAAs may lead to the enhancement of plant immunity.

It has been shown that plants respond to pathogens by enhanced activation of the phenylpropanoid pathway, which leads to the biosynthesis of phenolics, flavonoids, and isoflavonoids [21]. The antifungal activity of flavonoids in plants has been well documented [22,23]. In the metabolome, the phenylpropanoid biosynthesis pathway and the isoflavonoid biosynthesis pathway were significantly enriched (Figure 5C). Isoflavones are a class of phenolic compounds mainly restricted to the legume family, where they are involved in essential interactions with plant-associated microbes, including in defense against pathogens [24]. Vegetative stage shading (VS) increases isoflavone accumulation in soybean pods, which affects disease resistance [25]. Association analysis of the transcriptome and metabolome revealed that the most significantly enriched pathway was the isoflavonoid biosynthesis pathway (*p* < 0.05). Furthermore, the metabolomic data of isoflavone content were analyzed and annotated in metabolic pathways. The levels of isoflavonoids such as daidzein and malonylgenistin, which are located downstream of the isoflavone biosynthesis pathway, were significantly higher in the OE group than in the WT group. Therefore, the increase in isoflavone levels in OE plants during *P. sojae* infection may be a mechanism for enhancing soybean resistance to PRR.

KEGG analysis showed that the DEGs in WT24h vs. OE24h were enriched in the plant–pathogen interaction pathway. In the OE group, most of the DEGs in profile 4 were also involved in plant–pathogen interaction, suggesting that plant–pathogen interaction-related genes play an important role in the disease resistance of OE plants after *P. sojae* infection. Further analysis revealed that the above two groups of DEGs share 37 genes, including those encoding LRR and NB-ARC domain disease resistance protein, cyclic nucleotide-gated channel (CNGC) protein, calcium-binding protein, WRKY, and pathogenesis-related protein (Table S26). These genes could have a beneficial effect on soybean resistance to *P. sojae*. We further examined the expression patterns of *NLR* genes, and most of the *NLR* genes were significantly increased in the OE group at 36 hpi, indicating that the activation of *NLR* genes was more intense and persistent in OE compared to WT (Figure S10A). ETI activation triggers an increase in [Ca^2+^]_cyt_ and the initiation of downstream immune responses [26]. The calcium ion (Ca^2+^) is known to be a universally distributed intracellular second messenger in plants [27]. The cyclic nucleotide-gated channel has been proposed as a key Ca^2+^-conducting channel [28], and cyclic nucleotides have been implicated in plant responses to biotic and abiotic stresses [29]. In this study, DEGs encoding CNGCs showed significant increases in both groups, with even higher expression levels in WT plants (Figure S10B). We hypothesize that OE plants may activate other Ca^2+^ channels during *P. sojae* infection. The activated CC-NLR HOPZ-ACTIVATED RESISTANCE1 (ZAR1) forms a pentamer in the plasma membrane and acts as a Ca^2+^ channel to induce Ca^2+^ fluxes to stimulate cell death and ETI [26,30]. We examined the expression pattern of ZAR1 genes in the transcriptome and found that their expression was repressed in WT and up-regulated in OE at 36 hpi (Figure S10C). The helper NLRs, NRG1s, and ADR1s, which function downstream of TIR-NLRs, also form oligomers upon activation and function as Ca^2+^-permeable channels [31]. Whether *Glyma.18g283200* is involved in the activation of other *NLR* genes deserves further investigation.

In addition to PTI and ETI, systemic acquired resistance (SAR) plays an essential role in disease resistance and is modulated by signaling phytohormones such as jasmonic acid (JA), salicylic acid (SA), and abscisic acid (ABA) [32,33]. JA is a key phytohormone involved in developmental processes and plant defense responses [34]. The JA biosynthesis mutant *def1* is highly susceptible to two bacteria, two fungi, and one oomycete (*Phytophthora infestans*) [35]. Strawberry prime defense responses are mediated by the upregulation of several defense-related genes and maintain the upregulation of *MYC2* and *JAZ1* after MeJA treatment [36]. Genes encoding TIFY proteins and genes encoding the transcription factors MYC2 showed increased expression at the later stage of OE plant infection (Figure S11A). The JA signaling pathway may contribute to the induction of defense responses against *P. sojae*. Previous evidence suggests that brassinosteroid (BR) signaling plays a critical role in coordinating plant growth and immunity [37,38,39]. The GSK2-OsDLA-OsWRKY53 module works in tandem to synchronize the BR signaling with blast resistance in rice [40]. The changes in BR levels play an important role in mediating the defense response in soybean by regulating the expression patterns of R genes, especially NBS-LRR genes [41]. After BR is recognized by cell membrane-localized receptors, it undergoes a cascade of phosphorylation reactions to signal and inactivate the negative regulator BIN2 protein kinase, thereby deregulating downstream transcription factors and activating the BR response [42]. In this study, we found that the BR biosynthesis pathway genes were differentially expressed between the WT and OE groups (Figure S11B). Combined with the transcriptome data, it was hypothesized that under high BR levels, BIN2 (*Glyma.13G224300*) is inactivated in OE plants and its inhibition of BZR1/2 is released by triggering their dephosphorylation and translocation to the nucleus, where they bind to BR-responsive genes, inducing transcriptional reprogramming and thus modulating the defense response (Figure S11C).

## 4. Conclusions

Combined transcriptomic and metabolomic analysis provides an effective approach to discovering metabolic networks and key genes [43,44]. In the current study, the correlation between DEGs and DAMs was established, showing that the overexpression of the *NLR* gene could improve isoflavonoid biosynthesis in soybean. This study identified that isoflavonoids have the potential to be used as marker metabolites for PRR resistance in soybean. It is expected that with further quality breeding for improvement in isoflavones in soybean, PRR can be effectively controlled. In addition, it was speculated that ZAR1, valine, leucine, and isoleucine degradation, and plant hormone signal transduction may be involved in the defense response of soybean to *P. sojae*. However, the specific mechanism of disease resistance remains to be elucidated. In conclusion, this study provided an important resource for disease-resistance gene mining and is expected to provide a new theoretical basis for improvement and innovation in soybean genetic breeding.

## 5. Materials and Methods

### 5.1. Plant Materials, Inoculations, and Experimental Conditions

The soybean line Dongnong 50 (susceptible, WT) was obtained from the National Center for Soybean Improvement, Nanjing, Jiangsu Province, China, and used in this study. *P. sojae* isolate P7076 (vir. 1a, 1b, 1c, 1d, 1k, 3a) was obtained from the Department of Plant Pathology, Nanjing Agricultural University. In a previous study, the *Glyma.18g283200* overexpression line (resistant, OE) was generated and characterized using Donnong 50 as the transgenic receptor (Figure S2). At least 50 seeds of WT and OE lines were planted in sterilized vermiculite in plastic square basins (side length = 15 cm) and placed in the growth chamber at 25 °C with a 14 h light/10 h dark cycle. One-week-old seedlings were inoculated with *P. sojae* isolate P7076, which was grown on V8 juice agar plates (10% Campbell’s V8 vegetable juice, 0.02% CaCO_3_, 1.5% Bacto-agar) for 7 days using the hypocotyl inoculation method [13]. After inoculation, the seedlings were placed in a mist chamber (90% relative humidity) for 12 h and then transferred to a growth chamber at 25 °C with a 14 h light/10 h dark cycle. Hypocotyls were collected from inoculated seedlings at 12, 24, and 36 hpi by excising 2 to 3 cm across the wounded area and immediately being placed in liquid nitrogen before storage at −80 °C for subsequent experiments. Hypocotyl tissue from each of the three inoculated seedlings was mixed for one biological replicate. RNA sequencing was set up with three biological replicates per time point. Six biological replicates were set up for LC-MS/MS analysis.

### 5.2. RNA Extraction, Library Construction, and Sequencing

Total RNA was extracted using the TRIzol Reagent Kit (Invitrogen, Carlsbad, CA, USA) according to the manufacturer’s protocol, and genomic DNA was removed with DNase I (TaKaRa, Dalian, China). RNA quality analysis was performed using the Thermo Scientific NanoDrop One^C^ Spectrophotometer (Waltham, MA, USA). A high-quality RNA sample (OD260/280 = 1.8~2.2, OD260/230 ≥ 2.0, RIN ≥ 6.5, 28S:18S ≥ 1.0, >10 μg) was used to construct the sequencing library by using the NEBNext Ultra RNA Library Prep Kit (NEB #7530, New England Biolabs, Ipswich, MA, USA) for Illumina. After completing a polyA selection using oligo(dT) beads, mRNA was isolated and fragmented with a fragmentation buffer. Illumina’s protocol was followed for the cDNA synthesis, end repair, A-base addition, and ligation of the Illumina-indexed adapters. The cDNA libraries were then the size selected for 200–300 bp target fragments, followed by 15 cycles of PCR using Phusion DNA Polymerase (NEB M0530, New England Biolabs, Ipswich, MA, USA). The resulting cDNA library was sequenced using Illumina Novaseq6000 by Gene Denovo Biotechnology Co. (Guangzhou, China).

### 5.3. Differential Expression Analysis and Functional Enrichment

The differentially expressed genes (DEGs) were selected with the thresholds of log2 (fold-change) ≥ 1 and false discovery rate (FDR) < 0.05 using the edge package in R [16]. Gene Ontology (GO) enrichment analysis provides all GO terms that are significantly enriched in DEGs compared to the genome background and filters the DEGs corresponding to biological functions. All DEGs were mapped to GO terms in the Gene Ontology database (http://www.geneontology.org/, accessed on 14 February 2024). GO enrichment analysis was performed using the topGO package in R (corrected *p*-value < 0.05). Pathway enrichment analysis identified significantly enriched metabolic pathways or signal transduction pathways in DEGs compared to the whole genome background. The KEGG database (http://www.genome.jp/kegg, accessed on 14 February 2024) was used for pathway analysis [45].

### 5.4. Quantitative Real-Time Polymerase Chain Reaction Assays

DEGs were randomly selected from RNA-seq for RT-qPCR analysis, and the constitutively expressed *GmActin* (*Glyma.18g290800*) was employed as the internal reference gene. Six DEGs were selected for validation at each time point. The experiment was performed with three biological replicates, each with three technical replicates. All specific primer pairs are listed in the Supplementary Table S19. Template cDNAs were synthesized from 1.0 µg of total RNAs using the Prime Script™ RT Reagent Kit (TaKaRa, Kyoto, Japan) according to the protocol. ChamQ Universal SYBR qPCR Master Mix (Q711, Vazyme, Nanjing, China) was used as the labeling agent. Real-time PCR amplification and detection were performed on the Roche LightCycler^®^ 480 Instrument II (manufactured by F. Hoffmann-La Roche Ltd., Basel, Switzerland). The expression level of each target gene was determined using the 2^−ΔΔCt^ method [46].

### 5.5. Metabolites Extraction

The sample was transferred to an EP tube. After adding the extract solution (acetonitrile: methanol = 1:1, containing isotopically labeled internal standard mixture), the samples were vortexed for 30 s, sonicated in an ice water bath for 10 min, and incubated at −40 °C for 1 h to precipitate proteins. The sample was then centrifuged at 12,000× *g* rpm for 15 min at 4 °C. The resulting supernatant was transferred to a clean 2 mL LC/MS glass vial for the UHPLC-QE-MS analysis.

### 5.6. LC-MS/MS Analysis

LC-MS/MS analysis was conducted using a UHPLC system (Vanquish, Thermo Fisher Scientific, Waltham, MA, USA) with a UPLC BEH Amide column coupled to a Q Exactive HFX mass spectrometer (Orbitrap MS, Thermo Fisher Scientific, Waltham, MA, USA). The QE HFX mass spectrometer was utilized for its capability to acquire MS/MS spectra on information-dependent acquisition (IDA) mode under the control of acquisition software (Xcalibur, Thermo Fisher Scientific, Waltham, MA, USA). The acquisition software continuously analyzes the full-scan MS spectrum in this mode.

### 5.7. Screening and Identification of Differential Accumulation Metabolite (DAM)

Both multivariate and univariate statistical significance (VIP > 1.0 and *p* < 0.05) were used as the thresholds for the significance of differences in metabolite accumulation. Metabolites were annotated against several databases, including the Fiehn databases and the NIST 11 standard mass spectral databases, by comparing their mass spectra with those of authentic standards.

### 5.8. Integrated Metabolome and Transcriptome Analysis

Association analysis of the metabolome and transcriptome was performed by Omicsmart (https://www.omicsmart.com, accessed on 20 February 2024). Pearson correlation tests were used to detect associations between discriminant metabolite and discriminant gene expression content based on metabolite content and gene expression data. Three models, including the pathway functional model, the O2PLS model, and the correlation coefficient model were analyzed based on the gene expression and metabolite abundance values.

## Data Availability

All data supporting the findings of this study are available in the paper and Supplementary Materials.

## Supplementary Materials

The following supporting information can be downloaded at: https://www.mdpi.com/article/10.3390/plants13121705/s1, Figure S1. Soybean hypocotyl samples were collected from wild-type and the overexpression line infected with *P. sojae* at 12 hpi, 24 hpi, and 36 hpi. Seeds of the WT and OE lines were planted in sterilized vermiculite in a plastic square basin (side length = 15 cm) and placed in the growth chamber at 25 °C with a 14 h light/10 h dark cycle. One-week-old seedlings were inoculated with *P. sojae* strains P7076 by the injured hypocotyl inoculation method. Seedling hypocotyl samples were collected infected with *P. sojae* at 12 hpi, 24 hpi, and 36 hpi for RNA sequencing. Scale bar, 1 cm. Figure S2. Verification of *Glyma.18G283200* overexpression in transgenic soybean plants. (A) Identification of *Glyma.18G283200* transgenic positive plants by PCR validation. Identification of positive transgenic lines with specific primers using WT (lanes 1 to 3) and OE (lanes 4 to 8) plant genomic DNA as templates. Lanes 1, 4, and 7: Results of the 35S promoter primer and the Glyma.18G283200-Q-R primer PCR assay. Lanes 2, 5, and 8: Results of the NOS terminator primer and the Glyma.18G283200-Q-F primer PCR assay. Lanes 3, 6, and 9: PCR validation of the *bar* gene in the WT and OE plants. (B) RT-qPCR analysis of *Glyma.18G283200* transcripts in OE line. The allele of *Glyma.18G283200* in the WT (Dongnong 50), *SoyC12_18G282200* [47], was used to illustrate the relative expression levels of *Glyma.18G283200*. Amplification of soybean *GmActin11* was used as an internal control to normalize all data. Error bars represent the mean ± standard deviation of three biological replicates. * *p* < 0.05 indicates significant differences by Student’s *t*-test. Figure S3. Quality analysis of transcriptome sequencing between WT and OE. (A) Base composition and quality distributions. (B) Statistical maps of gene coverage. Figure S4. Correlation analysis between different samples. (A) Principal component analysis (PCA) of the similarities and differences between the eighteen samples used for RNA-seq in WT and OE plants after inoculation. (B) Pearson correlation coefficient analysis based on gene expression. Figure S5. The relative gene expression of randomly selected DEGs was examined by RT-qPCR and RNA-seq. Six DEGs were randomly selected to confirm their expression at 12 hpi (A), 24 hpi (C), and 36 hpi (E) by RT-qPCR. Amplification of soybean *GmActin11* was used as an internal control to normalize all data. Error bars represent the mean ± standard deviation of three biological replicates. * *p* < 0.05, ** *p* < 0.01 indicate significant differences by Student’s *t*-test. Heatmap of six DEGs from the RNA-seq data of 12 hpi (B), 24 hpi (D), and 36 hpi (F). Each column in the figure represents one sample, and each row represents one gene. The gene expression levels of the rows are normalized by z-scores. Red indicates increased gene expression; green indicates decreased gene expression. Figure S6. KEGG enrichment analysis of different enriched profiles. A. KEGG enrichment analysis of enriched profiles in the OE group. B. KEGG enrichment analysis of significantly enriched profiles in the WT group. Figure S7. OPLS-DA analysis. (A) OPLS-DA score diagram of POS mode. (B) OPLS-DA permutation test diagram of POS mode. (C) OPLS-DA load diagram of POS mode. (D) OPLS-DA score diagram of NEG mode. (E) OPLS-DA permutation test diagram of NEG mode. (F) OPLS-DA load diagram of NEG mode. Figure S8. Association analysis of transcriptome and metabolome. (A) Analysis of the common pathway of DEGs and metabolites. The y-axis represents the sum of the number of enriched genes and the number of enriched metabolites. (B) O2PLS load diagram. Transcriptome data are shown on the left and metabolome data on the right. Each point represents one gene or a metabolite. The x-axis is the first-dimensional coordinate of the joint part, and the y-axis is the second-dimensional coordinate of the joint part. The higher the correlation between an element and another omics, the higher the absolute value of the element in the coordinates. (C) Bipartite load diagram. (D) Heatmap of the correlation between gene expression and metabolite abundance. The heatmap was constructed using the FPKM value. Red and blue indicate positive and negative correlations, respectively. The asterisk indicates the presence of statistical significance for the correlation. * *p* < 0.05, ** *p* < 0.01, *** *p* < 0.001. (E) Network diagram correlating gene expression with metabolite abundance. Solid and dashed lines indicate positive and negative correlations, respectively. The line width indicates the degree of correlation. Figure S9. Heatmap of DEGs related to valine, leucine, and isoleucine degradation and the KEGG orthology map of amino acid biosynthesis. (A) Heatmap of the expression of genes related to the valine, leucine, and isoleucine degradation pathways in WT and OE plants inoculated with *P. sojae*. Gene expression of the rows was normalized by z-score; redder color indicates higher gene expression and greener color indicates lower gene expression. (B) KEGG orthology map (ko01230, biosynthesis of amino acids). The red circle-labeled metabolites are up-regulated, and the green circle-labeled metabolites are down-regulated. Figure S10. The plant–pathogen interaction pathways and heatmap of related DEGs. Heatmap of DEGs related to NLRs (A), CNGCs (B), and ZAR1 (C). Figure S11. The plant hormone signal transduction pathways and heatmap of related DEGs. (A) The JA signaling pathway and the heatmap of related DEGs. (B) The heatmap of DEGs enriched in BR biosynthesis. (C) The BR signaling pathway and the heatmap of related DEGs. Gene expression of the rows was normalized by z-score; redder color indicates higher gene expression and greener color indicates lower gene expression. The horizontal row represents a DEG with its gene ID, and the vertical columns represent WT_12 hpi, WT_24h hpi, WT_36h hpi, OE 12 hpi, OE 24 hpi, and OE 36 hpi from left to right. Table S1. Summary of base quality before and after filtering and the number of mapped reads to the reference genome. Table S2. WT 12 h vs. OE 12 h significantly different gene information. Table S3. WT 24 h vs. OE 24 h significantly different gene information. Table S4. WT 36 h vs. OE 36 h significantly different gene information. Table S5. Information of shared up-regulated DEGs at three time points. Table S6. Information of shared down-regulated DEGs at three time points. Table S7. KEGG enrichment analysis of DEGs at 12 hpi. Table S8. KEGG enrichment analysis of DEGs at 24 hpi. Table S9. KEGG enrichment analysis of DEGs at 36 hpi. Table S10. GO term analysis of up-regulated DEGs at 12 hpi. Table S11. GO term analysis of up-regulated DEGs at 24 hpi. Table S12. GO term analysis of up-regulated DEGs at 36 hpi. Table S13. KEGG enrichment analysis of up-regulated DEGs at 12 hpi. Table S14. KEGG enrichment analysis of up-regulated DEGs at 24 hpi. Table S15. KEGG enrichment analysis of up-regulated DEGs at 36 hpi. Table S16. KEGG enrichment analysis of profile 2 DEGs in OE group. Table S17. KEGG enrichment analysis of profile 4 DEGs in OE group. Table S18. KEGG enrichment analysis of profile 0 DEGs in WT group. Table S19. KEGG enrichment analysis of profile 3 DEGs in WT group. Table S20. KEGG enrichment analysis of profile 7 DEGs in WT group. Table S21. Information of the KEGG pathway interaction network in POS. Table S22. Information of the KEGG pathway interaction network in NEG. Table S23. Analysis of the common pathway of DEGs and metabolites. Table S24. Differential metabolite analysis using OPLS-DA in POS mode. Table S25. Differential metabolite analysis using OPLS-DA in NEG mode. Table S26. The shared DEGs in the plant–pathogen interaction pathway between WT24h vs. OE24h and OE profile 4. Table S27. The primer pairs were used in this study.
