# Supplementary material for: Integrated Transcriptome and Metabolome Analysis Reveals Molecular Mechanisms Underlying Resistance to Phytophthora Root Rot
Source: Plants (Basel). 2024 Jun 19;13(12):1705. doi: 10.3390/plants13121705 (PMC11207509; doi:10.3390/plants13121705)
Supplement: Supplementary file 1 [file plants-13-01705-s001.zip › Supplementary Figure S1-S11.pdf]

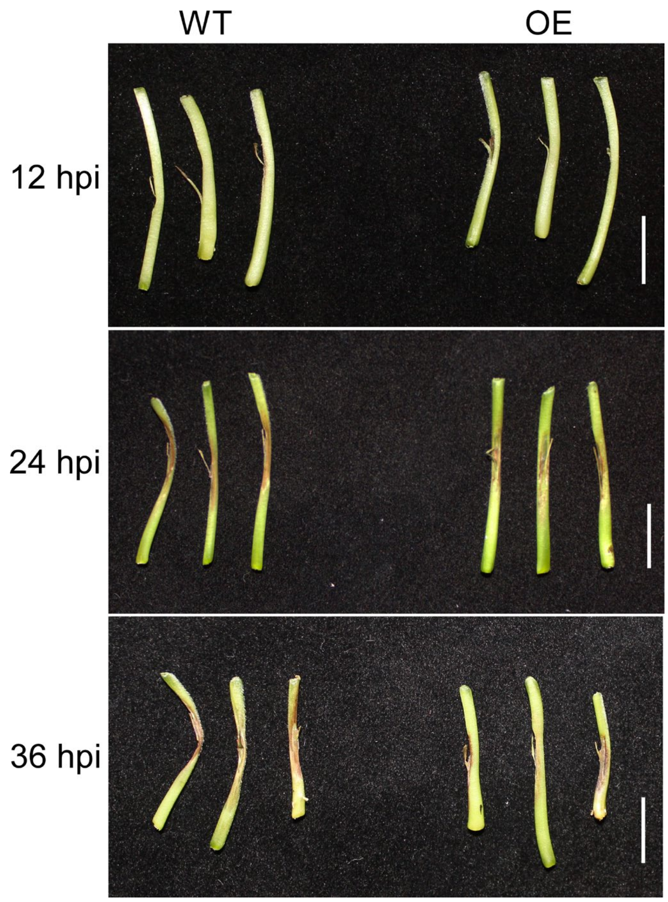

Figure S1. Soybean hypocotyl samples were collected from wild-type and the overexpression line infected with *P. sojae* at 12 hpi, 24 hpi, and 36 hpi.

Seeds of WT and OE line were planted in sterilized vermiculite in plastic square basin (side length = 15 cm) and placed in the growth chamber at 25°C with a 14 h light/10 h dark cycle. One-week-old seedlings were inoculated with *P. sojae* strains P7076 by injured hypocotyl inoculation method. Seedlings hypocotyl samples were collected infected with *P. sojae* at 12 hpi, 24 hpi, and 36 hpi for RNA sequencing. Scale bar, 1 cm.

A

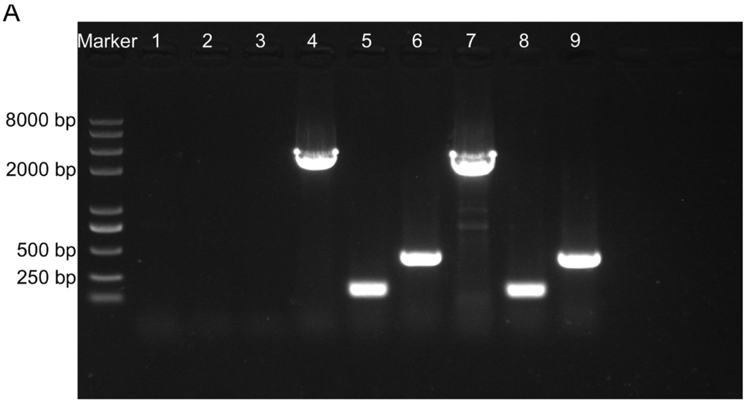

B

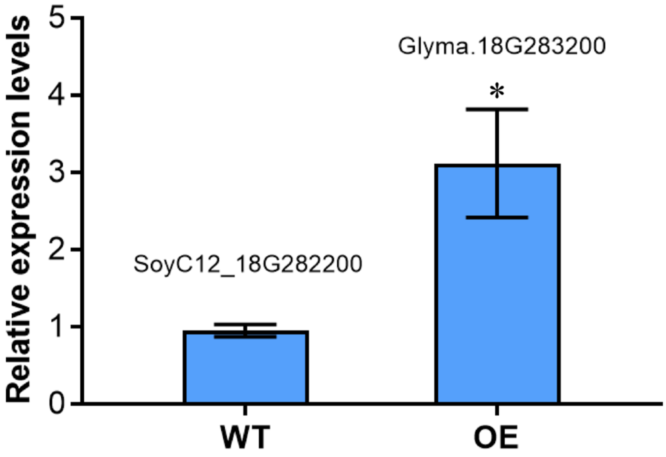

Figure S2. Verification of *Glyma.18G283200* overexpression in transgenic soybean plants.

(A) Identification of *Glyma.18G283200* transgenic positive plants by PCR validation. Identification of positive transgenic lines with specific primers using WT (lanes 1 to 3) and OE (lanes 4 to 8) plant genomic DNA as templates. Lanes 1, 4, and 7: Results of the 35S promoter primer and the Glyma.18G283200-Q-R primer PCR assay. Lanes 2, 5, and 8: Results of the NOS terminator primer and the Glyma.18G283200-Q-F primer PCR assay. Lanes 3, 6, and 9: PCR validation of the *bar* gene in the WT and OE plants. (B) RT-qPCR analysis of *Glyma.18G283200* transcripts in OE line. The allele of *Glyma.18G283200* in the WT (Dongnong 50), *SoyC12_18G282200* [50], was used to illustrate the relative expression levels of *Glyma.18G283200*. Amplification of soybean *GmActin11* was used as an internal control to normalize all data. Error bars represent the mean ± standard deviation of three biological replicates. *P < 0.05 indicate significant differences by Student's *t*-test.

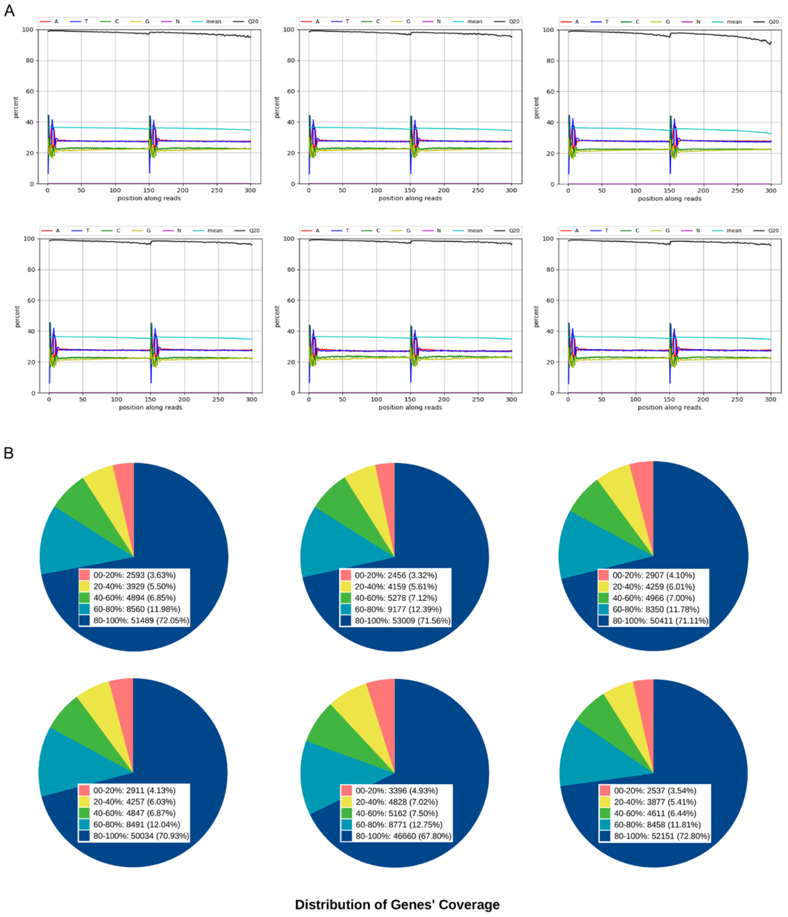

Figure S3. Quality analysis of transcriptome sequencing between WT and OE.

(A) Base composition and quality distributions. (B) Statistical maps of gene coverage.

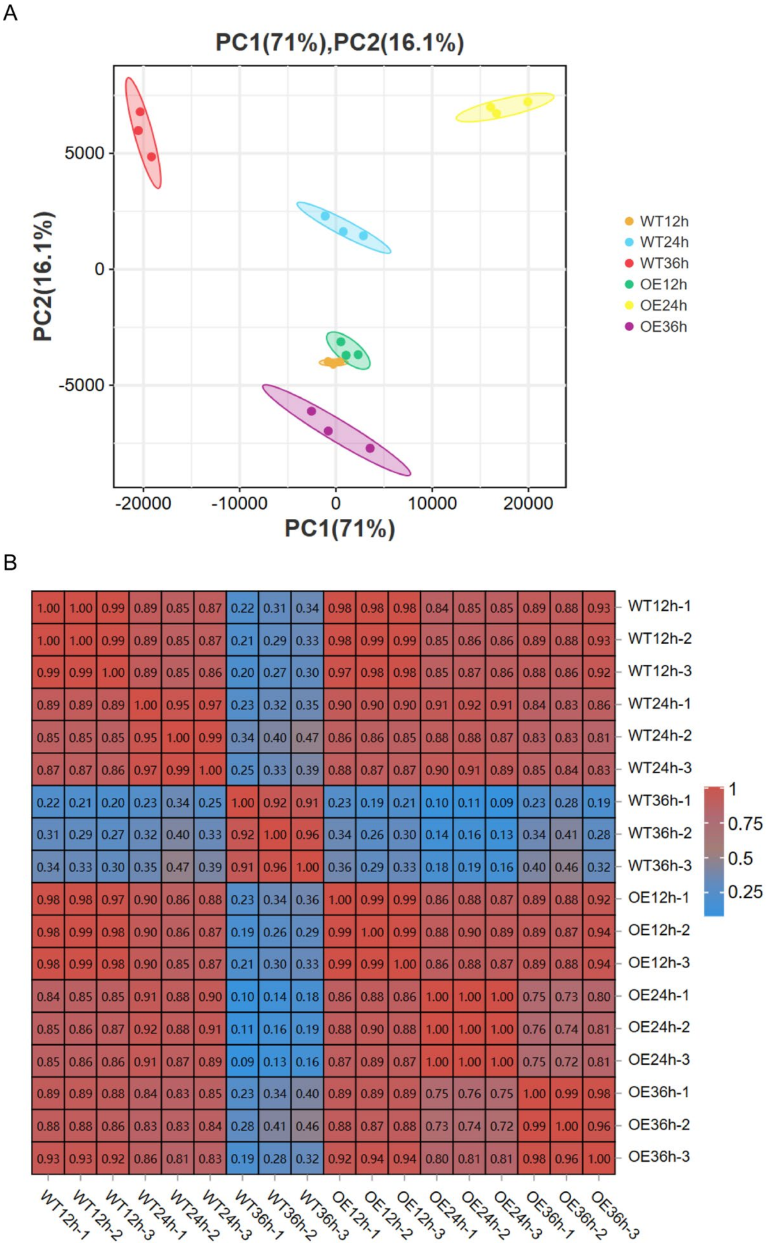

Figure S4. Correlation analysis between different samples.

(A) Principal component analysis (PCA) of the similarities and differences between the eighteen samples used for RNA-seq in WT and OE plants after inoculation. (B) Pearson correlation coefficient analysis based on gene expression.

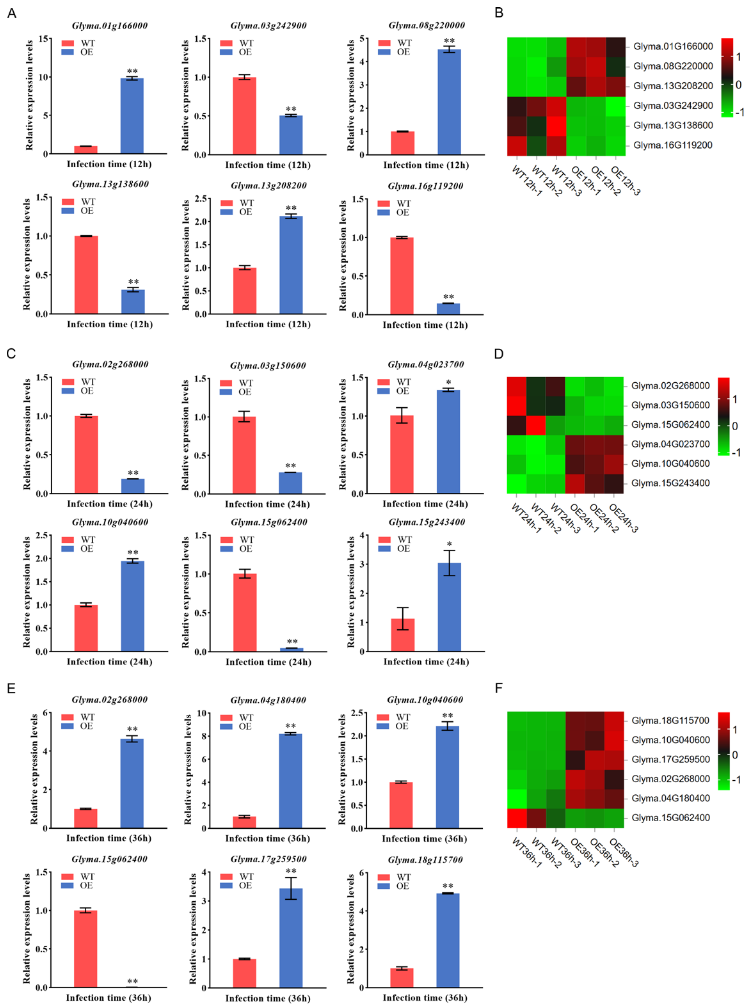

Figure S5. The relative gene expression of randomly selected DEGs was examined by RT-qPCR and RNA-seq.

Six DEGs were randomly selected to confirm their expression at 12 hpi (A): 24 hpi (C), and 36 hpi (E) by RT-qPCR. Amplification of soybean *GmActin11* was used as an internal control to normalize all data. Error bars represent the mean ± standard deviation of three biological replicates. **P* < 0.05, ***P* < 0.01 indicate significant differences by Student's *t*-test. Heatmap of six DEGs from the RNA-seq data of 12 hpi (B), 24 hpi (D), and 36 hpi (F). Each column in the figure represents one sample, and each row represents one gene. The gene expression levels of the rows are normalized by z-scores. Red indicates increased gene expression; green indicates decreased gene expression.

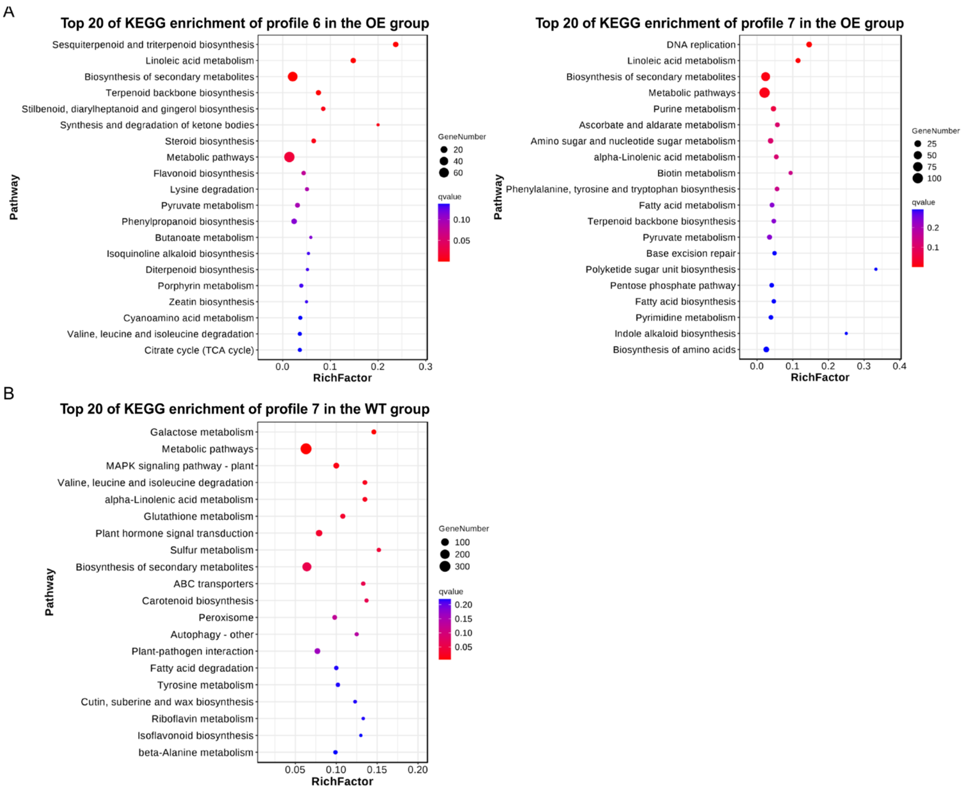

Figure S6. KEGG enrichment analysis of different enriched profiles.

A. KEGG enrichment analysis of enriched profiles in the OE group. B. KEGG enrichment analysis of significantly enriched profiles in the WT group.

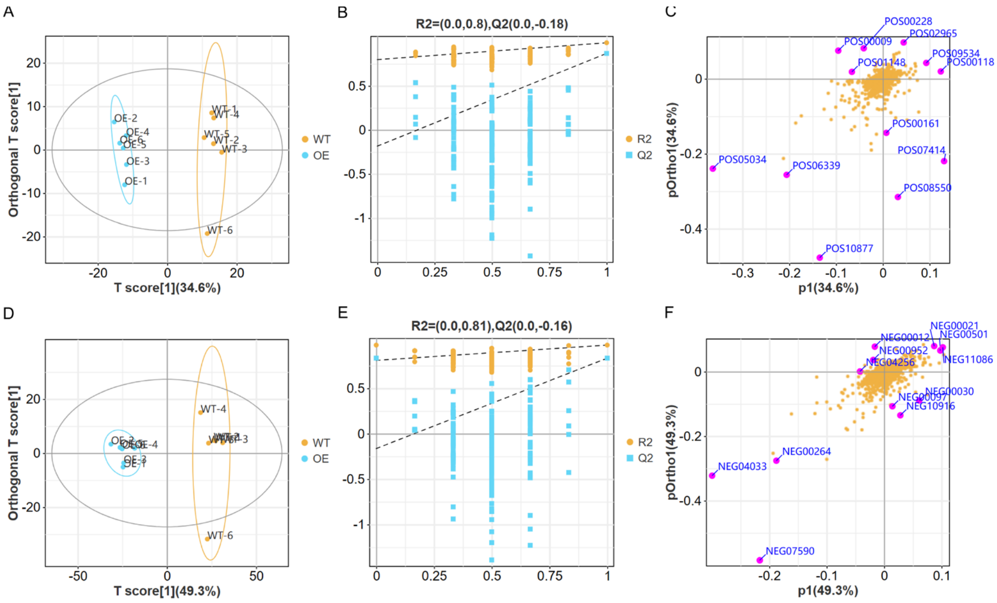

Figure S7. OPLS-DA analysis.

(A) OPLS-DA score diagram of POS mode. (B) OPLS-DA permutation test diagram of POS mode. (C) OPLS-DA load diagram of POS mode. (D) OPLS-DA score diagram of NEG mode. (E) OPLS-DA permutation test diagram of NEG mode. (F) OPLS-DA load diagram of NEG mode.

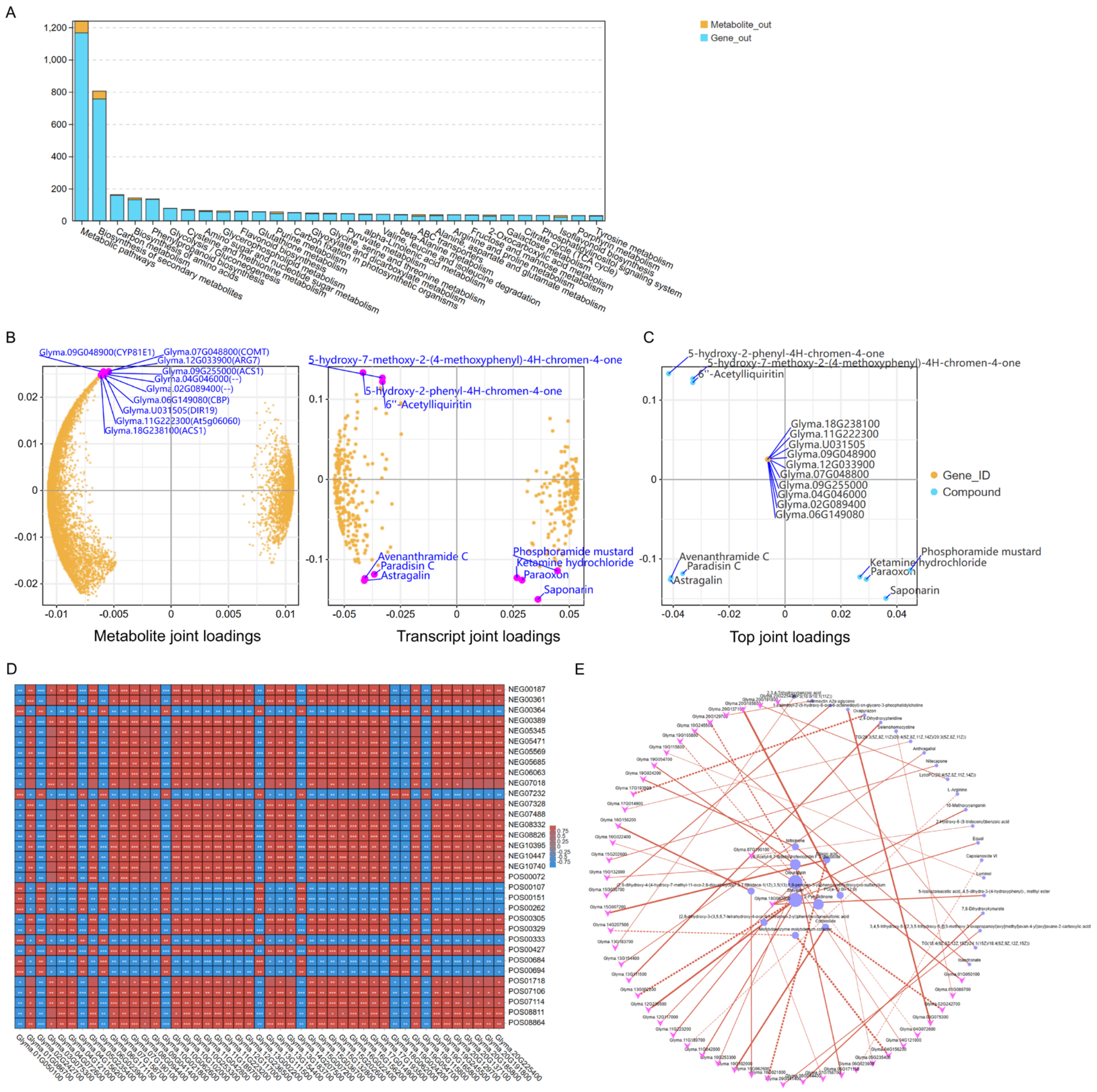

Figure S8. Association analysis of transcriptome and metabolome.

(A) Analysis of the common pathway of DEGs and metabolites. The y-axis represents the sum of the number of enriched genes and the number of enriched metabolites. (B) O2PLS load diagram. Transcriptome data are shown on the left and metabolome data on the right. Each point represents one gene or a metabolite. The x-axis is the first-dimensional coordinate of the joint part, and the y-axis is the second-dimensional coordinate of the joint part. The higher the correlation between an element and another omics, the higher the absolute value of the element in the coordinates. (C) Bipartite load diagram. (D) Heatmap of the correlation between gene expression and metabolite abundance. The heatmap was constructed using the FPKM value. Red and blue indicate positive and negative correlations, respectively. The asterisk indicates the presence of statistical significance for the correlation. *$P < 0.05$, **$P < 0.01$, ***$P < 0.001$. (E) Network diagram correlating gene expression with metabolite abundance. Solid and dashed lines indicate positive and negative correlations, respectively. The line width indicates the degree of correlation.

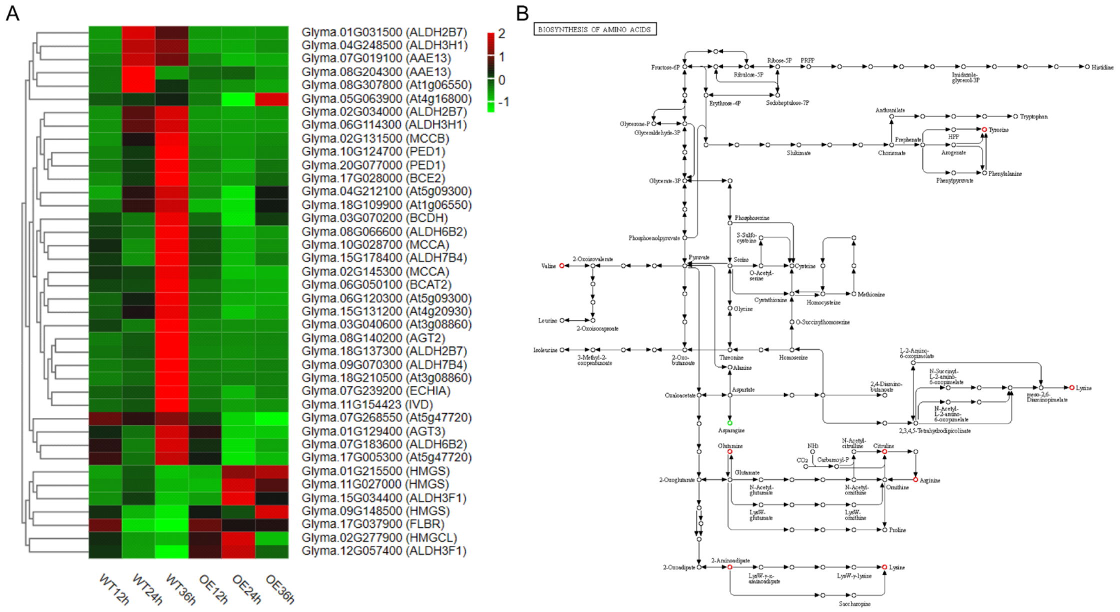

Figure S9. Heatmap of DEGs related to the valine, leucine and isoleucine degradation and KEGG orthology map of amino acids biosynthesis.

(A) Heatmap of expression of genes related to the valine, leucine and isoleucine degradation pathways in WT and OE plants inoculated with *P. sojae*. Gene expression of the rows was normalized by z-score; redder color indicates higher gene expression and greener color indicates lower gene expression. (B) KEGG orthology map (ko01230, Biosynthesis of amino acids). The red circle labeled metabolites are up-regulated, and the green circle labeled metabolites are down-regulated.

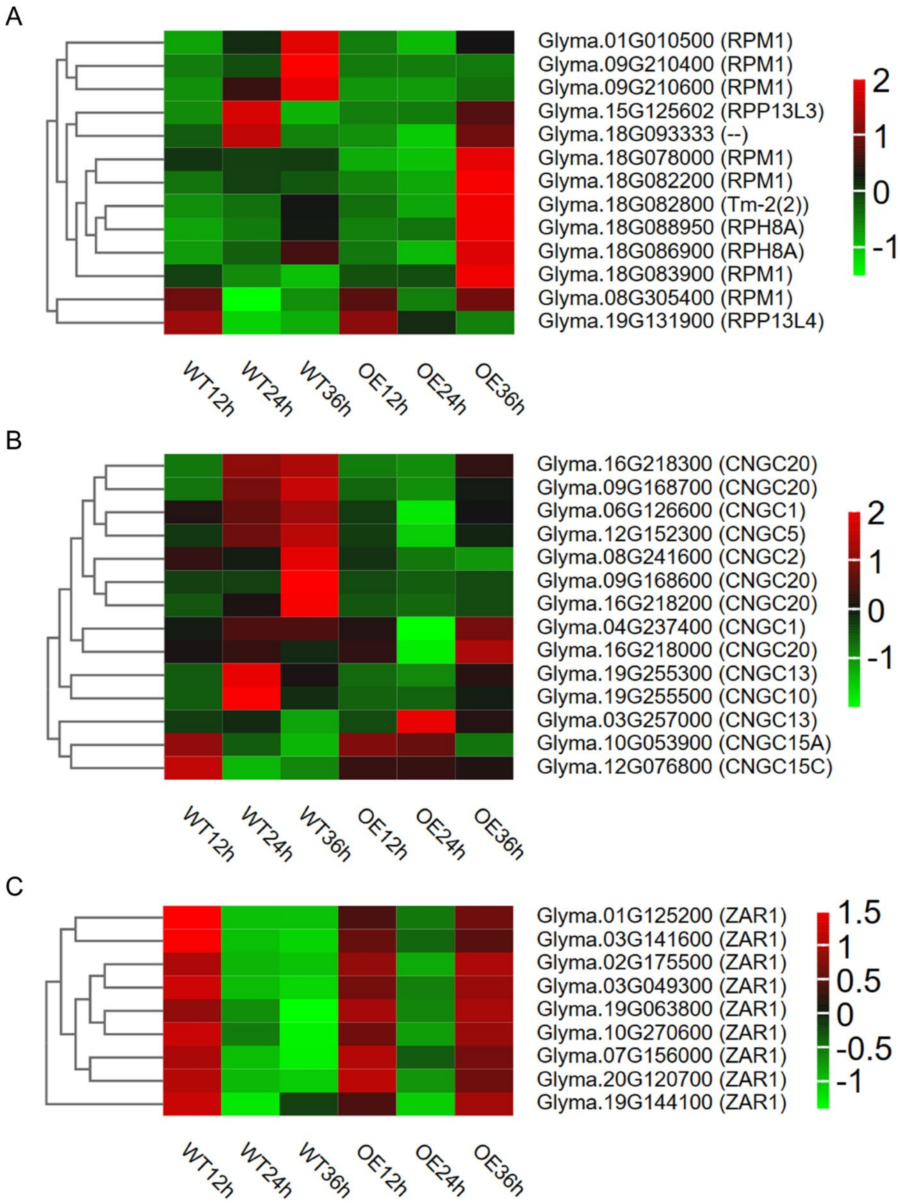

Figure S10. The plant-pathogen interaction pathways and heatmap of related DEGs.

Heatmap of DEGs related to NLRs (A), CNGCs (B), and ZAR1 (C).

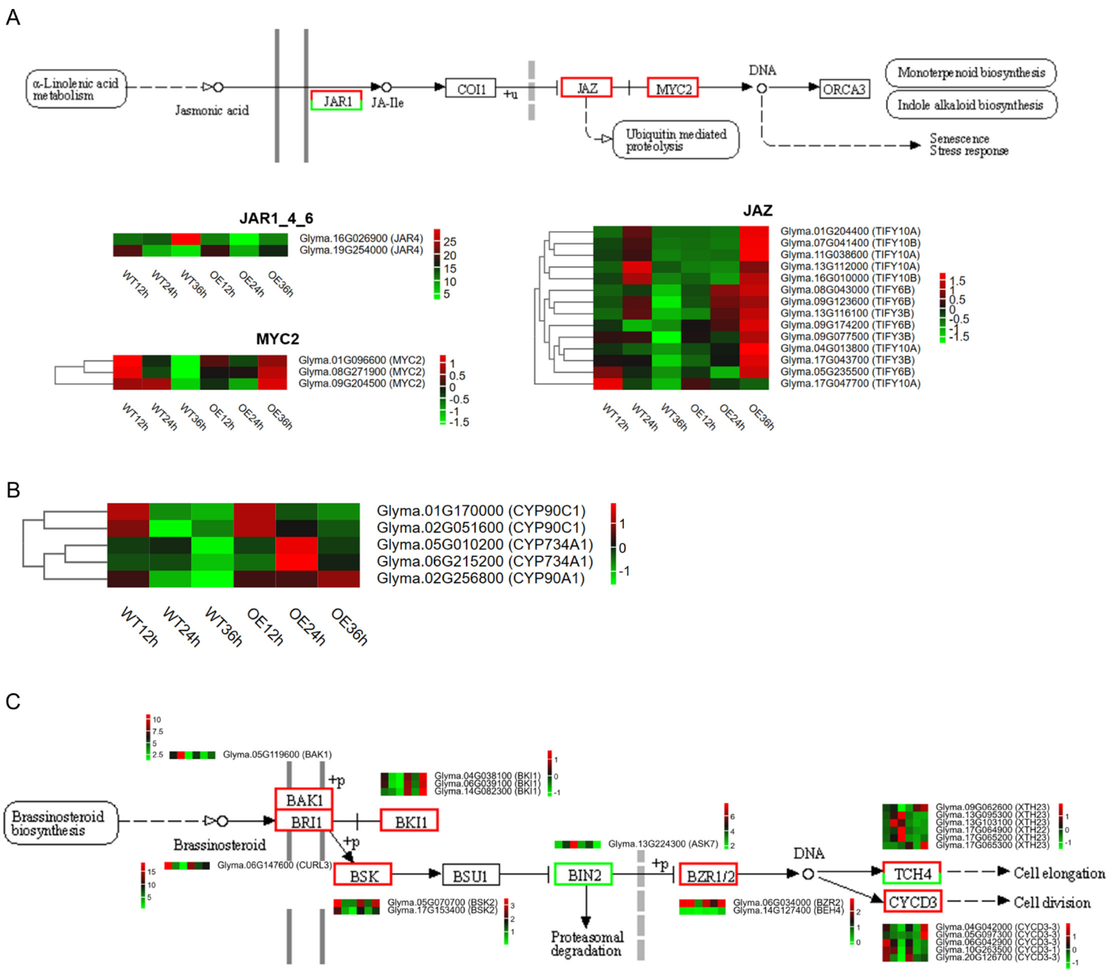

Figure S11. The plant hormone signal transduction pathways and heatmap of related DEGs.

(A) The JA signaling pathway and the heatmap of related DEGs. (B) The heatmap of DEGs enriched in BR biosynthesis. (C) The BR signaling pathway and the heatmap of related DEGs. Gene expression of the rows was normalized by z-score; redder color indicates higher gene expression and greener color indicates lower gene expression. The horizontal row represents a DEG with its gene ID, and the vertical columns represent WT_12 hpi, WT_24h hpi, WT_36h hpi, OE 12 hpi, OE 24 hpi, and OE 36 hpi from left to right.
